# The clinical risk and post-COVID-19 sequelae in patients with myasthenia gravis: a retrospective observational study

**DOI:** 10.3389/fneur.2025.1513649

**Published:** 2025-04-02

**Authors:** Chaoyue Zhang, Haocheng Luo, Yufei Deng, Hongjin Li, Xiang Yu, Jiaxin Liu, Linqi Huang, Xiaojun Yang, Qilong Jiang

**Affiliations:** ^1^The First Clinical Medical College of Guangzhou University of Chinese Medicine, Guangzhou, China; ^2^The First Affiliated Hospital of Chinese Medicine, Guangzhou University of Chinese Medicine, Guangzhou, China

**Keywords:** myasthenia gravis, persistent infection, coronavirus infections, post COVID-19 syndrome, sequelae

## Abstract

**Background:**

There are indeed several studies addressing the severity of Coronavirus disease 2019 (COVID-19) infection in myasthenia gravis (MG) patients. However, data on post-COVID-19 sequelae in MG patients remain limited. To address this gap, we collected clinical data on the condition and prognosis of MG patients with COVID-19 infection, aiming to investigate factors influencing both the severity of the infection and the occurrence of post-COVID-19 sequelae at 1 and 12 months after recovery.

**Method:**

This was a retrospective analysis of 150 MG patients with COVID-19 infection from November 1, 2022 to March 1, 2023 at the First Affiliated Hospital of Guangzhou University of Chinese Medicine, including patient demographics, clinical characteristics, and post-COVID-19 sequelae. Multivariable binary logistic and linear regression models were employed to ascertain the variables influencing the severity. The evolution of the post-COVID-19 sequelae was analyzed using McNemar's test.

**Result:**

In 150 MG patients, 128 (85.3%) patients were presented with COVID-19 infection, and 23 (18%) patients were hospitalized. The severity was associated with the daily corticosteroid dose (OR = 1.08, *p* = 0.02) and the frequency of myasthenia crises pre-COVID-19 (*b* = 7.8, *t* = 2.14, *p* = 0.035). Compared to normal patients, MG patients are more likely to experience post-COVID-19 sequelae such as insomnia, myalgia, dizziness, cough, expectoration, and sore throat at 12 months after recovery. Among these, the prevalence of myalgia, dizziness, rash, and vision impairment was significantly higher.

**Conclusion:**

Compared to normal patients, MG patients are prone to developing severe COVID-19 infection, which is associated with the daily corticosteroid dose and the frequency of myasthenia crises pre-COVID-19. Additionally, they are prone to experiencing post-COVID-19 sequelae, including insomnia, myalgia, dizziness, cough, expectoration, and sore throat, at 12 months after recovery.

## 1 Introduction

Coronavirus disease 2019 (COVID-19) was an acute infectious disease of the respiratory system caused by Severe Acute Respiratory Syndrome Coronavirus type 2 (SARS-CoV-2) (1, 2). As the virus continues to mutate, the development and refinement of outbreak prevention and control policies is an ongoing process. Following the introduction of the new policy in November 2022, COVID-19 spread rapidly, resulting in a new pandemic and once again attracting the attention of the general public, particularly those in at-risk groups. The outbreak was attributed to the Omicron variant strain, which exhibited a spectrum of clinical presentations from mild to severe. Common symptoms encompassed fever, dry cough, fatigue, nasal congestion, rhinorrhea, pharyngalgia, myalgia, and diarrhea. Severe cases often manifested with dyspnea and/or hypoxemia within about a week. In critical instances, the condition could rapidly deteriorate to encompass acute respiratory distress syndrome (ARDS), refractory metabolic acidosis, and multi-organ failure.

In addition to respiratory disease, COVID-19 has profound effects on a variety of organ systems over time. The cytokine storm induced by COVID-19 leads to excessive systemic inflammation and may increase the risk of neurological symptoms (including encephalitis and stroke) (3). In the neuromuscular system, a number of conditions have been observed, including cranial nerve palsies, peripheral nerve palsies, neuropathy, myalgia and Guillain-Barré syndrome (GBS) (4–7). The multi-sensory integration deficit, one of the most common sequelae of COVID-19 infection, is characterized by impairments in balance and proprioception (8). This condition may be linked to the neurotropism of SARS-CoV-2, the neuroinflammatory response triggered by COVID-19, and virus-induced myopathic changes. In patients with pre-existing neurological disorders such as myasthenia gravis (MG), this post-COVID clinical manifestation is significantly more severe and poses considerable management challenges.

Moreover, COVID-19 may exacerbate the health status of individuals with underlying neuromuscular disorders like myasthenia gravis (MG). This situation has generated concern among affected individuals and their healthcare providers about the risk of post-infection exacerbations or crises. MG patients, who are on long-term immunosuppressive therapy, exhibit a heightened vulnerability to adverse outcomes due to their immunocompromised state. Multiple studies have documented the impact of COVID-19 on patients with myasthenia gravis (MG), highlighting that the immunosuppressive therapy (IST) they receive poses a significant risk factor for the exacerbation of infections (9, 10). Conversely, Solé et al. (11) reported that immunosuppression was not an independent risk factor for exacerbating COVID-19 infection, although it showed significance in a univariate analysis. It has been proposed that the severity of COVID-19 infections in patients with myasthenia gravis is associated with the severity of myasthenia gravis itself rather than underlying chronic conditions. It has also been suggested that the severity of COVID-19 infections in myasthenia gravis patients is not related to the severity of myasthenia gravis itself, but rather to lung injury (12).

Our study aimed to delineate the clinical features of MG patients affected by COVID-19 in Guangdong and compare them with those normal individuals, in order to better elucidate the factors that impact the severity of COVID-19 infection and explore the post-COVID-19 sequelae in myasthenia gravis (MG) patients.

## 2 Methods

### 2.1 Study design and data extraction

This study was a single-center, bidirectional, observational clinical investigation that entailed the distribution of a questionnaire to myasthenia gravis (MG) patients from the patient database (*n* = 819) at the First Affiliated Hospital of Guangzhou University of Chinese Medicine. The primary objective was to collect data on COVID-19 infection among MG patients during the pandemic period from November 1, 2022, to March 1, 2023.

By March 1, 2023, a total of 150 questionnaires had been collected. Among the respondents, 128 patients had contracted COVID-19. Of these, 23 were hospitalized, 8 were admitted to the intensive care unit (ICU), and 2 died. At the meantime, we also integrate 450 normal individuals (non-MG individuals) who presented to the hospital after matching by age and sex, and collect their information related to COVID-19 infection. We found that 328 patients were infected and 3 required hospitalizations. For the 126 survivors' post-infection, we conducted follow-up surveys via telephone interviews and questionnaires to collect their post-COVID-19 sequelae at both 1- and 12-months post-recovery from the infection. Additionally, we administered a questionnaire to normal individuals (non-MG individuals) at 12-month post-recovery, and contrasted their responses with those of MG patients. A total of 2433 questionnaires were gathered.

**Figure 1:**
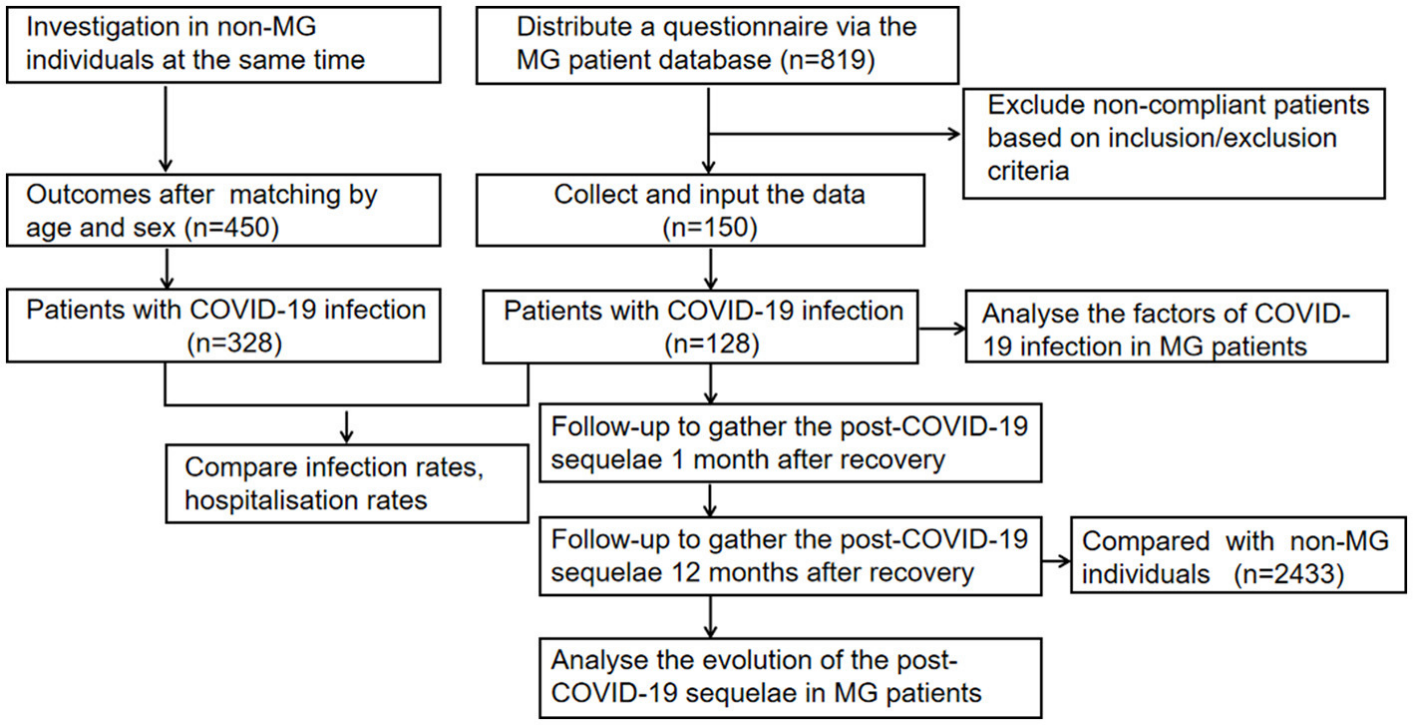
Study flowchart. MG, myasthenia gravis; COVID-19, coronavirus disease 2019.

The questionnaire for MG patients covers patient demographics, MG history [antibody status, thymus, frequency of myasthenic crises (MC) pre-COVID-19, current MG treatment], comorbidities, COVID-19 specifics [duration of fever, lengths of infection or hospitalization or ICU stay, daily corticosteroid (CS) dosage, vaccination status], and post-COVID-19 sequelae. And the questionnaire for non-MG individuals focuses on post-COVID-19 sequelae. Medical records were reviewed by three independent reviewers. Questionnaires, inpatient clinical notes and laboratory results were used by reviewers as primary data sources (Figure 1).

### 2.2 Participant selection (inclusion/exclusion criteria)

The inclusion criteria for this study were as follows: (1) Patients presenting with typical clinical manifestations of generalized myasthenia gravis (MG), characterized by fluctuating muscle weakness, were required to meet at least one of the following diagnostic criteria: (i) Positive pharmacological testing, defined as a positive response to the neostigmine methylsulfate test; (ii) Electrophysiological abnormalities, including a positive result on repetitive nerve stimulation (RNS) testing, characterized by a decremental response of >10% at low-frequency stimulation (3 Hz), or abnormal jitter and/or blocking on single-fiber electromyography (SFEMG); (iii) Seropositivity for anti-acetylcholine receptor (AChR) antibodies (13, 14). (2) Patients identified as positive for COVID-19 infection were verified through laboratory testing (nasopharyngeal polymerase chain reaction or antibody testing), clinical history, and chest imaging.

The exclusion criteria are: (1) Patients with incomplete information on COVID-19 diagnosis. (2) Patients without a documented history of myasthenia gravis (MG). (3) Patients lacking data on current MG-specific medication or those who could not be contacted.

### 2.3 Endpoints

Endpoints were COVID-19 infection, severity of COVID-19 and the post-COVID-19 sequelae at 1 or 12 months after “recovery.” The severity of COVID-19 course was classified as “non-hospitalized” (mild), “hospitalized without intensive care” (moderate), and “hospitalized with intensive care or deceased” (severe). The lengths of infection/hospital/ICU stay were also defined as secondary outcome measures of its severity. The definition of recovery encompasses either nucleic acid conversion or the day of discharge for hospitalized patients. Our study aimed to delineate the clinical features of MG patients affected by COVID-19, investigate the factors influencing the severity of infection and analyze the difference of the post-COVID-19 sequelae between MG patients and normal individuals.

### 2.4 Statistical analysis

We applied the Shapiro-Wilk normality test to all parameters. Numerical variables conforming to the normal distribution are described as mean and standard deviation, otherwise as the median and interquartile range (IQR). Categorical variables are described as *n* (%). Categorical data were analyzed by using Chi-squared test or Fisher's exact test, while numerical data using independent-*t* test or Mann–Whitney test. *P* values < 0.5 were considered significant. In addition, multivariable binary logistic or linear regression models were employed to ascertain the variables influencing the severity of COVID-19 infection. The chi-squared test was used to compare the prevalence of sequelae after COVID-19 infection in MG patients with normal individuals and to analyze whether there was a statistical difference between the two groups. The evolution of the post-COVID-19 sequelae was analyzed using McNemar's test (paired chi-square test) as well. Statistical analyses were performed using SPSS 21 and GraphPad prism 5.0.

### 2.5 Ethics approval

The research received approval from the Ethics Committee of the First Affiliated Hospital of Guangzhou University of Chinese Medicine (K-2024-124). Informed consent was acquired from all participants or their legal guardians before the survey.

## 3 Result

### 3.1 Demographics and characteristics

By March 1, 2023, 150 MG patients have been integrated into our study, and the characteristics of COVID-19 infection in MG patients were shown in Tables 1, 2. 128 (85.30%) MG patients were infected with COVID-19 in the period between 1 November, 2022, and 1 March, 2023. Among them, 23 patients (18%) were hospitalized, 8 (6.3%) were admitted into intensive care unit (ICU), and 2 (1.6%) died. We found statistically significant differences in BMI [22 (3.2) vs. 27 (6.3), *p* = 0.016] and comorbidities [44 (34.3%) vs. 14 (63.6%), *p* = 0.012] between MG patients with and without COVID-19 infection. There is no statistically significant difference in vaccination status [67 (52.3%) vs. 12 (54.5%), *p* = 0.855].

**Table 1:** Baseline characteristics of myasthenia gravis (MG) patients with or without COVID-19.

**Variables**	**Patients with COVID-19**	**Patients without COVID-19**	***P***
*N* (%)	128 (85.3%)	22 (14.7%)	
Sex male, *n* (%)	33 (25.8%)	7 (31.8%)	0.861
Age in years, mean (SD)	42 (14.3)	45 (18.0)	0.353
Age at onset in years, mean (SD)	34 (15.2)	41 (19.8)	0.271
BMI, mean (SD)	22 (3.2)	27 (6.3)	0.016^*^
Disease duration in years, median (IQR)	6 (3, 10)	5 (3, 7)	0.923
**Antibody, n (%)**			0.801
AChR-Ab	82 (64.0%)	13 (59.0%)	
EOMG (< 50)	68 (53.1%)	10 (45.5%)	
LOMG (≥50)	14 (10.9%)	3 (13.6%)	
MuSK-Ab	6 (4.6%)	0	
AChR-Ab, MuSK-Ab	3 (2.3%)	1 (4.5%)	
Seronegative	15 (11.7%)	2 (9.1%)	
Unknown	22 (17.1%)	6 (27.2%)	
**Thymus**, ***n*** **(%)**			0.903
Normal	47 (36.7%)	10 (45.5%)	
Thymic hyperplasia	41 (32.0%)	4 (18.1%)	
Thymoma	40 (31.2%)	8 (36.4%)	
Thymectomy, *n* (%)	63 (49.2%)	12 (54.5%)	0.998
**Frequency of MC pre-COVID-19**			0.477
≤ 2, *n* (%)	109 (85.2%)	20 (90.9%)	
≥ 3, *n* (%)	19 (14.8%)	2 (9.1%)	
Comorbidities, *n* (%)	44 (34.3%)	14 (63.6%)	0.012^*^
Hyperglycemia	11 (8.6%)	6 (27.2%)	0.409
Hyperlipidemia	7 (5.5%)	3 (13.6%)	0.876
Hypertension	10 (7.8%)	8 (36.4%)	0.217
Cardiovascular disease	3 (2.3%)	2 (9.1%)	0.366
COPD	6 (4.7%)	1 (4.5%)	0.411
Digestive disease	5 (3.9%)	2 (9.1%)	0.761
Urological disease	0	2 (9.1%)	0.999
Thyroid disease	16 (12.5%)	1 (4.5%)	0.375
Cancer	3 (2.3%)	1 (4.5%)	0.669
**Current treatment**, ***n*** **(%)**			0.824
Pyridostigmine	108 (84.4%)	19 (86.3%)	
No IST or only steroids	49 (38.3%)	8 (36.4%)	0.943
Standard IST (AZA, MMF, CSA, Tacrolimus)	55(43.0%)	10 (45.5%)	0.932
Rituximab	6 (4.7%)	1 (4.5%)	0.988
Vaccinated	67 (52.3%)	12 (54.5%)	

Data are presented as *n* (%), mean (SD) or median (IQR).

^*^P < 0.05 is significant.

COVID-19, coronavirus disease 2019; SD, standard deviation; IQR, interquartile range; BMI, body mass index; AChR, acetylcholine receptor; MuSK, muscle-specific kinase; EOMG, early-onset myasthenia gravis; LOMG, late-onset myasthenia gravis; MC, myasthenia crisis; COPD, chronic obstructive pulmonary disease; IST, immunosuppressive therapy; AZA, azathioprine; MMF, mycophenolate mofetil; CSA, cyclosporin A.

**Table 2:** Characteristics of severity in MG patients with COVID-19.

**MG characteristics *n* (%), mean (SD) or median (IQR)**	**Non-hospitalized patients**	**Hospitalized patients**	**Hospitalized patients without intensive care**	**Hospitalized patients with intensive care**	**Deceased patients**
*N*	105 (82.0%)	23 (18.0%)	13 (10.2%)	8 (6.3%)	2 (1.6%)
Sex male	29 (27.6%)	4 (17.3%)	2 (15.3%)	2 (25.0%)	0
Age in years	41 (14.2)	45 (14.9)	45 (17.6)	47 (11.6)	35 (1.41)
Age at onset in years	33 (15.4)	37 (14.0)	36 (16.6)	39 (11.3)	32 (2.12)
BMI	22 (20, 25)	21 (20, 23)	21 (20, 23)	21 (20, 23)	22 (1.1)
Disease duration in years	5 (3, 10)	7 (2, 10)	4 (2, 14)	8 (5, 12)	4 (0.7)
**Antibody**					
AChR-Ab	68 (64.7%)	14 (60.8%)	7 (53.8%)	5 (62.5%)	2 (100.0%)
EOMG (< 50)	58 (55.2%)	10 (43.5%)	5 (38.5%)	3 (37.5%)	2 (100.0%)
LOMG (≥50)	10 (9.5%)	4 (17.3%)	2 (15.3%)	2 (25.0%)	0
MuSK-Ab	6 (5.7%)	0	0	0	0
AChR-Ab, MuSK-Ab	3 (2.8%)	0	0	0	0
Seronegative	11 (10.4%)	4 (17.3%)	2 (15.3%)	2 (25.0%)	0
Unknown	17 (16.1%)	5 (21.7%)	4 (30.7%)	1 (12.5%)	0
**Thymus**					
Normal	38 (36.1%)	9 (39.1%)	6 (46.1%)	3 (37.5%)	0
Thymic hyperplasia	33 (31.4%)	8 (34.7%)	4 (30.7%)	4 (50.0%)	0
Thymoma	34 (32.3%)	6 (26%)	3 (23.0%)	1 (12.5%)	2 (100.0%)
Thymectomy	52 (49.5%)	11 (47.8%)	5 (38.5%)	4 (50.0%)	2 (100.0%)
**Frequency of MC pre-COVID-19**					
≤ 2	91 (86.7%)	18 (78.3%)	12 (92.4%)	4 (50.0%)	1 (50.0%)
≥3	14 (13.3%)	5 (21.7%)	1 (7.6%)	4 (50.0%)	1 (50.0%)
Comorbidities	34 (32.3%)	10 (43.5%)	4 (30.7%)	4 (50.0%)	2 (100.0%)
Hyperglycemia	7 (6.6%)	4 (17.3%)	2 (15.3%)	2 (25.0%)	1 (50.0%)
Hyperlipidemia	5 (4.7%)	2 (8.6%)	2 (15.3%)	0	0
Hypertension	8 (7.6%)	2 (8.6%)	1 (7.6%)	1 (12.5%)	0
Cardiovascular disease	0	3 (13%)	2 (15.3%)	1 (12.5%)	0
COPD	4 (3.8%)	2 (8.6%)	1 (7.6%)	1 (12.5%)	0
Digestive disease	3 (2.8%)	1 (4.3%)	0	0	0
Urological disease	0	0	0	0	1 (50.0%)
Thyroid disease	14 (13.3%)	2 (8.6%)	1 (7.6%)	1 (12.5%)	0
Cancer	2 (1.9%)	1 (4.3%)	1 (7.6%)	0	0
**Current MG treatment**					
Pyridostigmine	87 (82.8%)	21 (91.3%)	12 (92.4%)	8 (100.0%)	2 (100.0%)
No IST or only steroids	38 (36.1%)	11 (47.8%)	6 (46.1%)	3(37.5%)	2 (100.0%)
Standard IST (AZA, MMF, CSA, and Tacrolimus)	48 (45.7%)	7 (30.4%)	3 (23.0%)	4 (50.0%)	0
Rituximab	4 (3.8%)	2 (8.6%)	2 (15.3%)	0	0
**Vaccination status**					
Vaccinated	58 (55.2%)	9 (39.1%)	7 (53.8%)	2 (25.0%)	0
Duration of fever, Days	3 (4.04)	3 (0, 5)	3 (1, 5)	5 (10.4)	9 (0.7)
Length of infection stay, days	11 (9.07)	15 (9, 30)	15 (7, 30)	19 (12, 30)	36 (0.7)
Length of hospital stay, days	NA	13 (6, 29)	10 (9.1)	32 (15.2)	16 (0.7)
Length of ICU stay, Days	NA	NA	NA	13 (7.1)	26 (0.7)
Daily CS dosage (mg)	10 (5, 18)	10 (5, 28)	14 (12.3)	17 (10.3)	11 (0.7)

Data are presented as *n* (%), mean (SD) or median (IQR).

COVID-19, coronavirus disease 2019; SD, standard deviation; IQR, interquartile range; BMI, body mass index; AChR, acetylcholine receptor; MuSK, muscle-specific kinase; EOMG, early-onset myasthenia gravis; LOMG, late-onset myasthenia gravis; MC, myasthenia crisis; COPD, chronic obstructive pulmonary disease; IST, immunosuppressive therapy; AZA, azathioprine; MMF, mycophenolate mofetil; CSA, cyclosporine A; ICU, intensive care unit; CS, corticosteroid.

In the meantime, we also investigated normal patients (non-MG patients) who came to the hospital at that period. A total of 450 patients were included after matching for age and sex. Of these, 328 (72.9%) patients were infected with COVID-19, 3 (0.9%) patients were hospitalized, and no patients were admitted to ICU or died.

We found statistically significant differences in the rates of infection [128 (85.3%) vs. 328 (72.9%), *p* < 0.05], hospitalization [23 (18%) vs. 3 (0.9%), *p* < 0.05], or mortality [2 (1.6%) vs. 0 (0%), *p* < 0.05] between MG and normal patients. Compared with normal patients, MG patients are more likely to develop infections and have more severe symptoms.

### 3.2 The severity of COVID-19 infection in MG patients

In order to investigate the factors influencing the severity of COVID-19 infection in MG patients, we included a subset of all MG patients affected by COVID-19 in further analyses (*n* = 128). According to the definition of severity, they were classified as non-hospitalized patients (105, 82.0%), hospitalized patients without intensive care (13, 10.2%), hospitalized patients with intensive care (8, 6.3%) and deceased patients (2, 1.6%). The average length of infection/hospital/ICU stay were also recorded, which have been shown in Table 2.

Compared with non-hospitalized patients' group, hospitalized patients' group has fewer male patients (17.3% vs. 27.6%), a higher percentage of patients who were positive for acetylcholine receptor antibodies (AChR-Ab) with late-onset MG (LOMG) (17.3% vs. 9.5%), more patients with the frequency of myasthenia crisis (MC) pre-COVID-19 ≥3 (21.7% vs. 13.3%), and a higher frequency of comorbidities (43.5% vs. 32.3%). Several known risk factors for poor outcome after COVID-19 were more frequent in the group of hospitalized or deceased patients: hyperglycemia (17.3% vs. 6.6%), hyperlipidemia (8.6% vs. 4.7%), COPD (8.6% vs. 3.8%), and cancer (4.3% vs. 1.9%). Both of 2 deceased patients had comorbidities: one with hyperglycemia and the other with a urological disease.

A multivariate binary logistic regression model was constructed to investigate the risk factors for the severity of COVID-19 infections. The dependent variable was whether MG patients were hospitalized after infection, and the model included age, sex, BMI, duration of disease, MG treatment, number of MC pre-COVID-19, thymus status, comorbidities, and daily CS dosage as independent variables. The results indicated that daily CS dosage was a risk factor for hospitalization (OR = 1.08, 95% CI 1.01–1.15, *p* = 0.02), suggesting that an increase in daily CS dosage would elevate the risk of hospitalization (Table 3a). A multivariable linear regression model was constructed to analyze the length of infection stay in MG patients, with adjustments made for age, sex, BMI, disease duration, MG treatment, number of myasthenic crises pre-COVID-19, thymus status, comorbidities, daily corticosteroid dosage, and vaccination status. The results revealed a statistically significant association between having three or more myasthenic crises pre-COVID-19 and a longer infection stay compared to those with two or fewer crises (*b* = 7.88, *t* = 2.14, *p* = 0.035). Furthermore, vaccination status was found to have no significant association with the length of infection stay (Table 3b).

**Table 3a:** Multivariable binary logistic regression models for risk of hospitalization in MG patients with COVID-19.

**Variables**	**β coefficient**	***P***	**OR (95% CI) for hospitalization**
Age at onset in years	0.03	0.123	1.03 (0.99–1.07)
Sex	0.61	0.378	1.84 (0.48–7.08)
Disease duration in years	0.04	0.303	1.04 (0.97–1.11)
BMI	−0.12	0.210	0.89 (0.74–1.07)
No IST or only steroids	−0.80	0.413	0.45 (0.07–3.03)
Standard IST	−1.51	0.148	0.22 (0.03–1.70)
CS dosage (mg)	0.07	0.022^*^	1.08 (1.01–1.14)
Frequency of MC pre-COVID-19 ≥3	0.81	0.228	2.25 (0.60–8.36)
Thymectomy	−0.11	0.845	0.90 (0.30–2.67)
Comorbidities	−0.31	0.643	0.74 (0.20–2.67)

^*^P < 0.05 is significant.

MG, myasthenia gravi;, OR, odds ratio; CI, confidence interval; COVID-19, coronavirus disease 2019; BMI, body mass index; MC, myasthenia crisis; IST, immunosuppressive therapy; CS, corticosteroid.

**Table 3b:** Multivariable linear regression models for risk of the length of infection stay in MG patients with COVID-19.

**Variables**	**β coefficient**	**Standard Error**	***t*-value**	***P***
Age at onset in years	0.16	0.10	1.67	0.098
Sex	−0.24	3.03	−0.08	0.938
Disease duration in years	−0.09	0.18	−0.53	0.597
BMI	−0.21	0.40	−0.53	0.598
No IST or only steroids	1.54	4.45	0.35	0.730
Standard IST	−0.90	4.52	−0.20	0.842
CS dosage (mg)	0.24	0.16	1.57	0.120
Frequency of MC pre-COVID-19 ≥3	7.88	3.68	2.14	0.035^*^
Thymectomy	−3.03	2.62	−1.16	0.249
Comorbidities	−1.66	3.05	−0.54	0.588
Vaccinated	−0.47	2.58	−0.18	0.856

^*^P < 0.05 is significant.

MG, myasthenia gravis; COVID-19, coronavirus disease 2019; BMI, body mass index; MC, myasthenia crisis; IST, immunosuppressive therapy; CS, corticosteroid.

### 3.3 The post-COVID-19 sequelae

To further investigate the residual symptoms of COVID-19 infection after nucleic acid conversion in MG patients and to compare them with normal patients. We included a subset of all surviving MG patients affected by COVID-19 (*n* = 126).

The results of the 1-month follow-up after nucleic acid conversion showed that 54 (42.9%) patients' symptoms of the infection had completely disappeared and their health status had returned to that before the infection, 17 (13.5%) felt that their symptoms of MG were still worse than before, and 72 (57.1%) patients had one or more post-COVID-19 sequelae. The most common symptoms were anxiety or insomnia (31, 24.6%), cognition impairment (29, 23%), vision impairment (24, 19.0%), sore throat (20, 15.9%) and cough (20, 15.9%). In addition, there are some other residual symptoms such as myalgia (17, 13.5%), expectoration (14, 11.1%), palpitation (9, 7.1%), ageusia (8, 6.3%), anosmia (5, 4.0%), dizziness (6, 4.8%), dyspnea (7, 5.5%), anorexia (12, 9.5%), depilation (16, 12.7%), tinnitus (9, 7.1%), and rash (1, 0.8%).

The results of the 12-month follow-up after nucleic acid conversion showed that 45% patients still have the post-COVID-19 sequelae. Of which symptoms such as vision impairment (42, 33.3%), myalgia (37, 29.3%), cognition impairment (25, 19.8%), and anxiety or insomnia (21, 16.7%) are relatively common.

**Figure 2:**
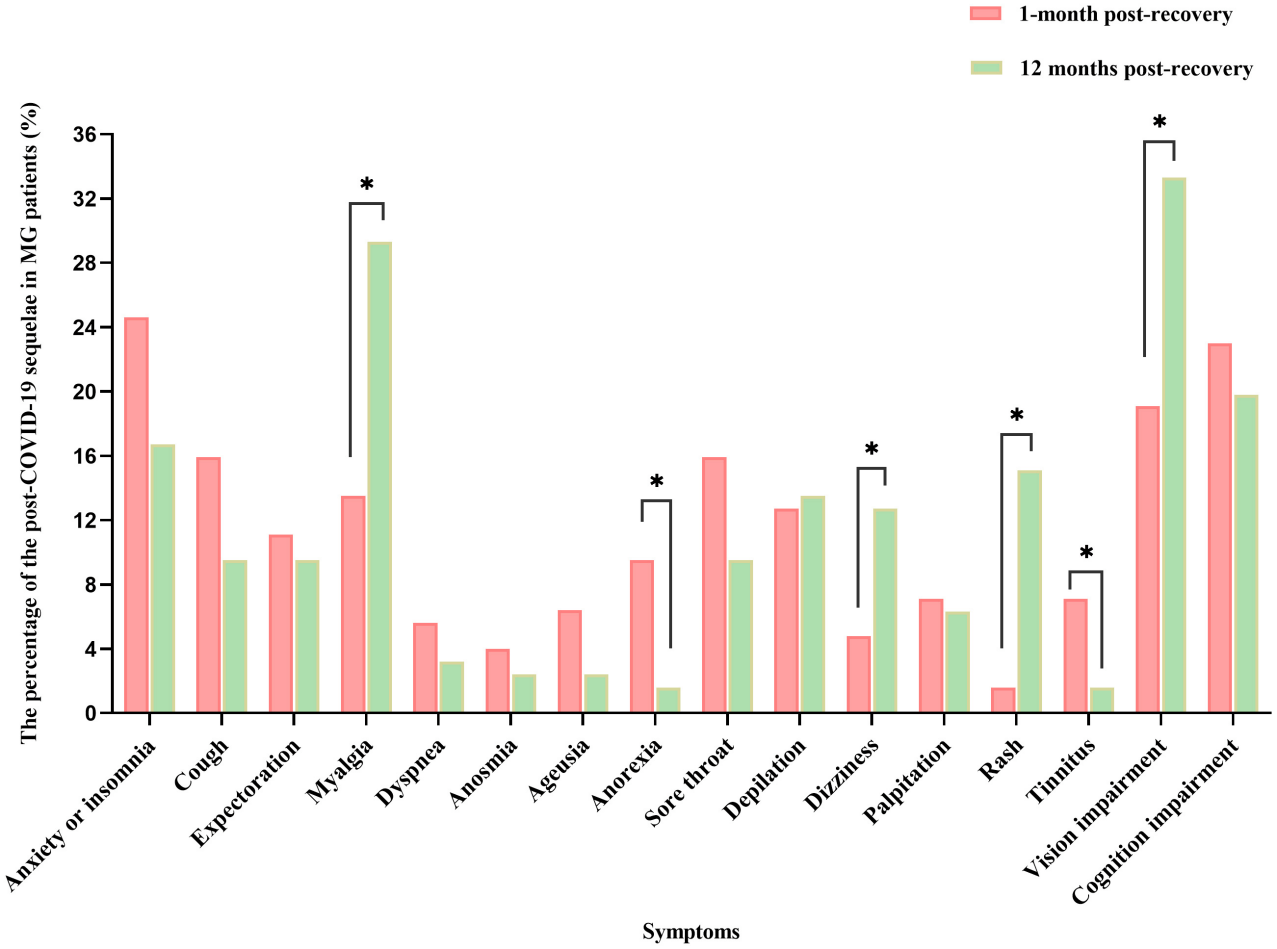
Comparison of the post-COVID-19 sequelae between 1-month and 12-month follow-up after nucleic acid conversion. *P < 0.05 is significant. MG, myasthenia gravis; COVID-19, coronavirus disease 2019.

A comparison of symptoms such as anxiety or insomnia, cough, expectoration, dyspnea, taste changes, impaired sense of smell, sore throat, depilation, palpitations, and impaired cognition at the 1-month and 12-month follow-ups showed no statistically significant differences. Compared to the results of the 12-month follow-up, there is a higher frequency of anorexia (9.5% vs. 1.6%, *p* < 0.05) and tinnitus (13.5% vs. 29.3%, *p* < 0.05) observed during the 1-month period following nucleic acid conversion according to scientific standards. In contrast, the frequency of myalgia (13.5% vs. 29.3%, *p* < 0.05), dizziness (4.8% vs. 12.7%, *p* < 0.05), rash (1.6% vs. 15.1%, *p* < 0.05), vision impairment (19.1% vs. 33.3%, *p* < 0.05) were higher in the 12-month period following nucleic acid conversion (Table 4, Figure 2).

**Table 4:** Comparison of the post-COVID-19 sequelae between 1-month and 12-month follow-up after nucleic acid conversion.

**Symptoms**	**MG patients (survivors)**, ***n*** **(%)**		***P***	**OR (95% CI) for hospitalization**
	**1-month post-recovery**	**12 months post-recovery**		
Anxiety or insomnia	31(24.6%)	21 (16.7%)	0.076	2.25 (0.98–5.17)
Cough	20 (15.9%)	12 (9.5%)	0.152	2.00 (0.86–4.67)
Expectoration	14 (11.1%)	12 (9.5%)	0.832	1.20 (0.52–2.78)
Myalgia	17 (13.5%)	37 (29.3%)	< 0.001^*^	11.00 (2.59–46.78)
Dyspnea	7 (5.6%)	4 (3.2%)	0.250	4.00 (0.45–35.79)
Anosmia	5 (4.0%)	3 (2.4%)	0.625	3.00 (0.31–28.84)
Ageusia	8 (6.4%)	3 (2.4%)	0.061	Infinity
Anorexia	12 (9.5%)	2 (1.6%)	< 0.001^*^	11.00 (1.42–85.20)
Sore throat	20 (15.9%)	12 (9.5%)	0.169	1.89 (0.84–4.24)
Depilation	16 (12.7%)	17 (13.5%)	1.000	1.13 (0.43–2.92)
Dizziness	6 (4.8%)	16 (12.7%)	< 0.001^*^	11.00 (1.42–85.20)
Palpitation	9 (7.1%)	8 (6.3%)	1.000	1.20 (0.37–3.93)
Rash	2 (1.6%)	19 (15.1%)	< 0.001^*^	18.00 (2.40–134.84)
Tinnitus	9 (7.1%)	2 (1.6%)	0.039^*^	8.00 (1.00–63.97)
Vision impairment	24 (19.1%)	42 (33.3%)	< 0.001^*^	19.00 (2.54–141.93)
Cognition impairment	29 (23%)	25 (19.8%)	0.329	1.19 (0.67–2.13)

^*^P < 0.05 is significant.

MG. myasthenia gravis; COVID-19, coronavirus disease 2019; OR, odds ratio; CI, confidence interval.

**Figure 3:**
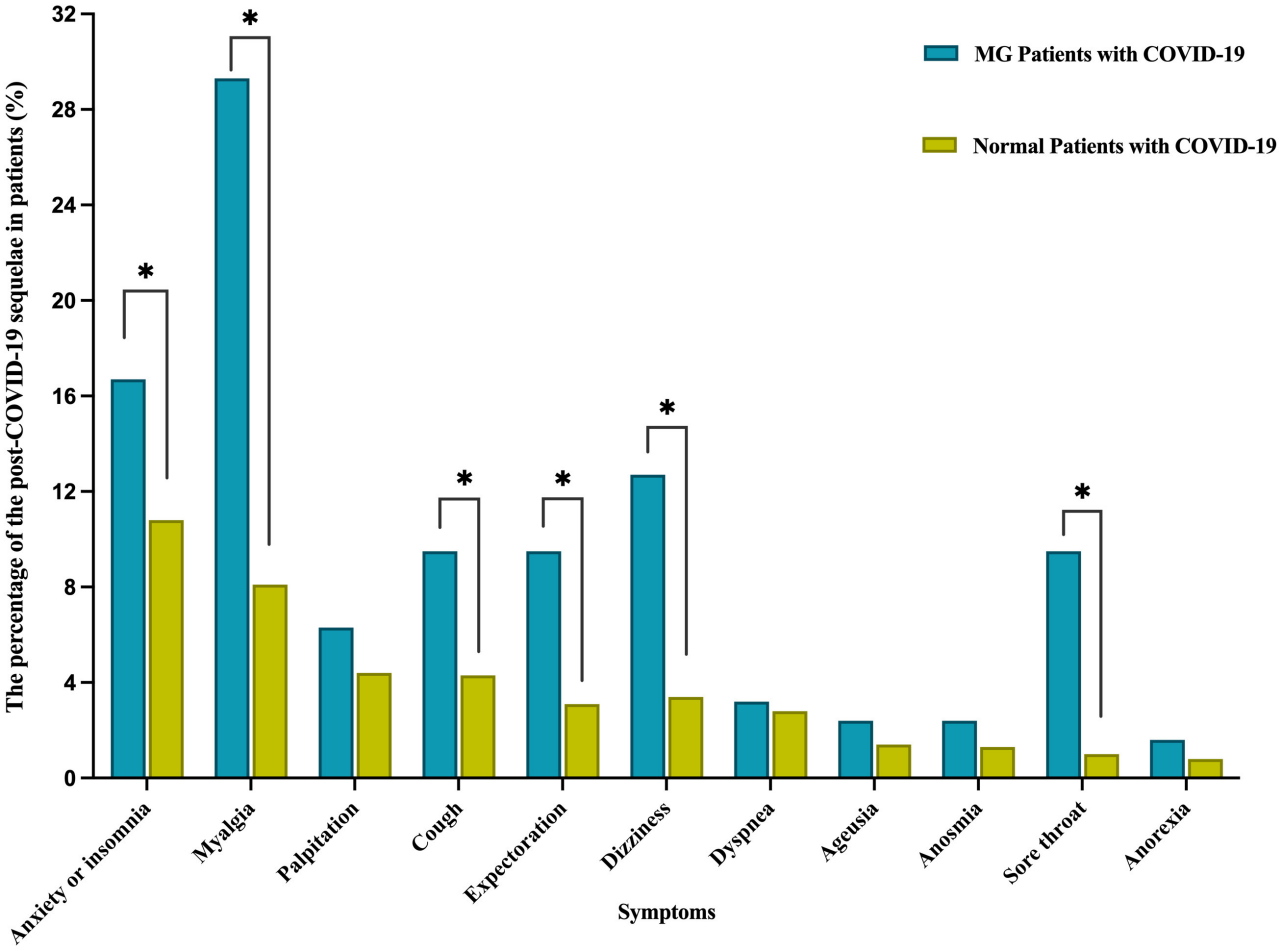
Comparison of the post-COVID-19 sequelae between MG Patients and Normal Patients at 12-month follow-up after nucleic acid conversion. *P < 0.05 is significant. MG, myasthenia gravis; COVID-19 coronavirus disease 2019; Anxiety or insomnia was statistically significant (*p* = 0.04).

Upon comparing the 12-month follow-up results of patients with MG (*n* = 126) to those of normal individuals (*n* = 2,433), it was observed that the MG group had a significantly higher prevalence of several residual symptoms (Table 5, Figure 3). These symptoms included anxiety or insomnia (16.7% vs. 10.8%, *p* < 0.05), myalgia (29.3% vs. 8.1%, *p* < 0.05), cough (9.5% vs. 4.3%, *p* < 0.05), expectoration (9.5% vs. 3.1%, *p* < 0.05), dizziness (12.7% vs. 3.4%, *p* < 0.05), and sore throat (9.5% vs. 1.0%, *p* < 0.05). While the incidence of palpitations, dyspnoea, taste change, impaired sense of smell, and anorexia was higher in the MG group, there was no statistically significant difference between the two groups (*p* > 0.05).

**Table 5:** Comparison of the post-COVID-19 sequelae between MG patients and Normal Patients at 12-month follow-up after nucleic acid conversion.

**Symptoms**	**Patients (survivors)**, ***n*****(%)**		***X* ^2^**	***P***
	**MG patients with COVID-19 (*****n*** = **126)**	**Normal patients with COVID-19 (*****n*** = **2,433)**		
Anxiety or insomnia	21 (16.7%)	262 (10.8%)	4.24	0.040^*^
Myalgia	37 (29.3%)	198 (8.1%)	64.72	< 0.001^*^
Palpitation	8 (6.3%)	106 (4.4%)	1.12	0.290
Cough	12 (9.5%)	104 (4.3%)	7.63	0.006^*^
Expectoration	12 (9.5%)	75 (3.1%)	13.24	< 0.001^*^
Dizziness	16 (12.7%)	82 (3.4%)	25.83	< 0.001^*^
Dyspnea	4 (3.2%)	69 (2.8%)	0.00	1.000
Ageusia	3 (2.4%)	35 (1.4%)	0.23	0.635
Anosmia	3 (2.4%)	32 (1.3%)	0.37	0.541
Sore throat	12 (9.5%)	25 (1.0%)	54.87	< 0.001^*^
Anorexia	2 (1.6%)	20 (0.8%)	0.17	0.680

^*^P < 0.05 is significant.

MG, myasthenia gravis; COVID-19 coronavirus disease 2019.

## 4 Discussion

### 4.1 The risk of infection

We found statistically significant differences in the rates of infection (85.3% vs. 72.9%, *p* < 0.05), hospitalization (18% vs. 0.9%, *p* < 0.05), or mortality (1.6% vs. 0%, *p* < 0.05) between MG patients and normal patients. Compared with normal patients, MG patients are more likely to develop infections and have more severe symptoms.

On the one hand, this phenomenon can be attributed to adjustments made to pertinent prevention strategies, resulting in a significant and swift rise in the overall number of COVID-19 infections nationwide. These adjustments may include policies such as the discontinuation of quarantine measures for infected individuals by the National Health and Health Commission (NHHC). On the other hand, the immune-compromised state of the MG population, stemming from prolonged oral administration of hormones and other immunosuppressants, renders them more vulnerable to novel coronavirus infections and may result in more pronounced symptoms. In cases where necessary, these individuals may require adjunctive ventilatory support. Therefore, caution should continue to be exercised when treating MG patients during novel coronavirus infections.

### 4.2 The severity of infection

Our data indicates that the daily CS dosage taken prior to infection by MG patients is a significant risk factor for COVID-19-related hospitalizations. Moreover, higher daily CS doses are associated with an increased likelihood of requiring hospitalization due to infections, consistent with findings from a study conducted by Michala Jakubíková et al. (15). And in New York, patients with immune-mediated inflammatory diseases who were treated with chronic corticosteroids were found to have a higher likelihood of being hospitalized for COVID-19 compared to those who were not receiving CS treatment (16).

The reasons are as follows: (1) long-term oral CS treatment suppresses T-cell activation and differentiation and result in lymphopenia (17). (2) The use of CS seemed to protract SARS-CoV-2 viral clearance, leading to an extended period of viremia, and exacerbate the myasthenia gravis particularly during the early stages of infection (18). (3) Unstable MG patients with large dosage of CS have faster deterioration of respiratory parameters during COVID-19 infection.

However, in our study, neither treatment with corticosteroids alone nor standard Immunosuppressive Therapy (IST) regimens were identified as significant risk factors for the duration of infection in myasthenia gravis (MG) patients. Additionally, the use of immunosuppressant was not found to be associated with the duration of infection in this population. This is consistent with the findings of Sole et al. (11). One plausible explanation for these findings could be the potential beneficial effects of certain immunotherapies in mitigating COVID-19 infection. These therapies may offer protection by modulating the exaggerated inflammatory response, such as the cytokine storm, which is often linked to the worsening of clinical outcomes in COVID-19 patients (3). It is possible that these immunotherapies could act to suppress immune cell migration and reduce chemokine production during the later stages of infection, potentially dampening the secondary immune response. Current guidelines recommend that in the event of COVID-19 infection in myasthenia gravis patients, existing immunosuppressive medications are generally considered safe and should not be abruptly discontinued.

The study data also indicated that patients with three or more MC pre-COVID-19 experienced longer durations of infection compared to those with two or fewer. This observation suggests the importance of early identification of patients with multiple MC, ongoing monitoring to prevent crises, timely intervention, proactive use of ventilatory support, and readiness for adjunctive treatments such as plasma exchange and intravenous immunoglobulin (IVIg) if necessary.

Our findings indicate that vaccination status is not associated with the length of infection stay in MG patients. This aligns with the results of Ari Breiner's study, which demonstrated that pre-infection vaccination does not exacerbate the condition of MG patients, and patients generally tolerate the vaccine well (19). Furthermore, several studies support the recommendation to promote vaccination for MG patients, especially during the ongoing COVID-19 pandemic (20).

### 4.3 The post-COVID-19 sequelae

It is well known that COVID-19 causes multi-organ dysfunction during acute infections, and some patients even develop long-term symptoms called the post-COVID-19 sequelae. A cohort study of 6-month consequences of COVID-19 in patients discharged from hospital showed that at 6 months after acute infection, COVID-19 survivors were mainly troubled with fatigue or muscle weakness, sleep difficulties, and anxiety or depression (21). SARS-CoV-2 RNA has been shown to be widely distributed throughout the body and can persist *in vivo* for months (22). Although the respiratory tract is the most common site of persistence of SARS-CoV-2 RNA, ≥50% of advanced cases also have persistent RNA in the myocardium, head and neck lymph nodes, thoracic cavity, sciatic nerves, ocular tissues, and all sampled areas of the central nervous system except the dura mater. This presents compelling evidence to elucidate the sequelae in systems beyond the respiratory system. Our study reports on residual symptoms observed at 1 and 12 months post nucleic acid conversion in MG patients who had confirmed COVID-19 infection, juxtaposed with residual symptoms noted in normal individuals post recovery.

#### 4.3.1 MG patients with normal patients

The main strength of our study is that it compares the sequelae of infections in patients with myasthenia gravis with the sequelae in normal subjects over the same period. Compared to normal individuals, MG survivors infected with COVID-19 were significantly more likely to have certain clinical symptoms 12 months after nucleic acid conversion. This applies not only to respiratory symptoms, but also to other systemic and neuropsychological symptoms including insomnia or anxiety, myalgia, dizziness, cough, expectoration and sore throat. An observational study of survivors after COVID-19 infection showed that ~55% of patients had no residual symptoms at 12 months (full recovery to pre-covid-19 status), which is consistent with the results of our study that ~45% of patients had the post-COVID-19 sequelae (23).

SARS-CoV-2 is neurotoxic (24). Neurological symptoms led by insomnia and anxiety are one of the aggregated manifestations of COVID-19. The pandemic has had a significant negative impact on mental health, with studies indicating that individuals who have recovered from COVID-19 often experience long-term psychiatric symptoms. These symptoms include post-traumatic stress disorder (PTSD), depression, anxiety, insomnia, and obsessive-compulsive disorder (OCD). The mental health issues observed in individuals who have not contracted the virus may be attributed to a variety of factors, such as the direct experience of the SARS-CoV-2 infection, the effects of isolation, the practice of physical distancing, incomplete recovery of physical health, economic challenges, and the psychological impact of being aware of COVID-19 related fatalities (25, 26).

MG patients exhibit more pronounced symptoms of anxiety and insomnia after recovery compared to the general population. This may be attributed to the long-term use of corticosteroids and other immunosuppressive agents, which place them in a state of immunosuppression. Consequently, they may have heightened concerns about the potential for recurrent respiratory distress, myasthenic crises, and life-threatening conditions due to infections. We hypothesize that in older adults with long-standing myasthenia gravis (MG), prolonged infection with SARS-CoV-2 and sustained inflammatory responses could exert enduring detrimental effects on the central nervous system.

The occurrence of myalgia following COVID-19 may be associated with inflammatory responses, where the levels of pro-inflammatory cytokines are elevated, thereby playing a role in muscle damage. In addition to myalgia, COVID-19 is also associated with myositis which is induced by direct muscle infection, and it could trigger an autoimmune response (5). The severity of this condition can vary widely, including generalized muscle weakness, back pain, dermatomyositis, rhabdomyolysis, and paraspinal pain. Firstly, SARS-CoV-2 may infect the central nervous system through hematogenous spread and retrograde neural pathways, including via the vagus nerve from the lungs (27, 28). Additionally, the virus could alter blood-brain barrier permeability, facilitating cytokine entry into the CNS and promoting neuroinflammation. This may explain the high prevalence of neuroinflammation, neuromuscular disease in covid-19 patients, and may lead to devastating long-term effects (29).

Myalgia may also be associated with “post-COVID sarcopenia.” Studies have shown that post-COVID syndrome is strongly related to a loss of muscle mass and function, and these patients often exhibit multisensory integration deficits (30). This aligns with our findings that MG patients are prone to myalgia and dizziness 12 months after recovery.

Our research indicates that myalgia, as a common symptom of myasthenia gravis itself, is more likely to persist, worsen, or recur after the acute phase of COVID-19 compared to the general population. Furthermore, there is a difference in the incidence of myalgia one month and 12 months after recovery from the virus, with a higher incidence observed at the 12-month follow-up. We speculate about the potential mechanisms of myalgia could be the reactivation of latent autoimmune responses. On the one hand, as the most common antibody in MG, the receptor of AChR-Ab shares structural similarities with the SARS-CoV-2 receptor, potentially leading to the reactivation of latent autoimmune responses. In MG patients infected with COVID-19, the robust immune response to the virus may also increase antibodies targeting the AChR, exacerbating clinical symptoms of MG and potentially leading to increased myalgia or disease relapse. On the other hand, some studies have reported associations between MuSK antibody-associated MG and SARS-CoV-2. Given the molecular differences, the underlying mechanism for MuSK-related MG is more likely a breakdown of self-tolerance rather than cross-reactivation (31, 32).

Therefore, following viral infection, it is essential to promptly assess the antibody levels in patients, particularly in those who are AChR-Ab positive. The decision to initiate immunotherapy should be based on a comprehensive evaluation of the severity of MG symptoms and the degree of myalgia exacerbation, with the aim of reducing pathogenic antibody levels (33). In MG patients who develop severe immune responses (e.g., cytokine storm) following COVID-19 infection, early initiation of immunotherapy, such as tocilizumab (TCZ) or convalescent plasma (CP) therapy, may be considered. Monoclonal antibodies like TCZ are particularly suitable for managing severe inflammatory responses associated with COVID-19, such as elevated IL-6 levels. CP therapy is recommended for critically ill patients, especially those with high viral loads. However, for patients with mild symptoms, early use of immunotherapy is generally not advised to avoid unnecessary adverse effects. Furthermore, given the inherent immune dysregulation in MG patients, the initiation of immunotherapy requires careful assessment of the patient's immune status and potential risks. It is recommended to closely monitor immune markers (e.g., IL-6, CRP) during the early stages of infection and to collaborate with specialists in neurology, infectious diseases, and immunology to develop individualized treatment plans.

In MG patients, COVID-19 infection may exacerbate the Tregs/IL-17 imbalance and induce an increase in circulating anti-AChR antibody levels, potentially leading to residual respiratory issues later on. However, in our study, the proportion of MG patients with dyspnea was not significantly different from that of the normal patients, which may be related to the activation of certain physiological adaptation mechanisms to avoid fatigue, mainly by central neural output inhibition of the respiratory muscles leading in acute hypoventilation and hypercapnia (34, 35). Some researchers define this phenomenon as silent or “happy” hypoxemia, where MG patients infected with SARS-CoV-2 experience reduced respiratory muscle load, thereby exhibiting milder respiratory distress or none that is distinguishable from the normal patients (36).

#### 4.3.2 The evolution of the post-COVID-19 sequelae in MG patients

The post-COVID-19 sequelae is characterized as a relapsing-remitting condition. Our research has identified three distinct patterns in the progression of symptoms over time. Initially, we observed that the prevalence of symptoms such as anorexia and tinnitus decrease over time, being more common in the short term than 12 months after nucleic acid conversion (recovery). Subsequently, the prevalence of certain symptoms increased over time. For instance, myalgia showed an increase from 13.5% (1-month post-recovery) to 29.3% (12 months post-recovery). Other symptoms, including dizziness, rash, and vision impairment exhibited a similar trend of increasing prevalence. Ultimately, symptoms with stable prevalence over time may be driven by mechanisms that do not rapidly change with time, such as insomnia, palpitations, cognition impairment, cough, expectoration, dyspnea, sore throat, anosmia, ageusia, and depilation. These may be a result of a combination of acute disease recovery and the delayed sequelae of COVID-19.

## 5 Advantages and limitations

To our awareness, few studies have investigated the longitudinal evolution of the post-COVID-19 sequelae, which predominantly focusing on the general population. Our study is the first to report the major clinical sequelae and their trends in the MG population in the short- and long-term following recovery from COVID-19 infection, and to compare the long-term sequelae with those in the general population. However, there were also some limitations.

Firstly, compared to face-to-face communication or physical examinations, the nature of questionnaires or telephone follow-ups may result in lower accuracy of information obtained in this study. Secondly, the study enrolled a relatively small number of MG patients, and most of them were not hospitalized or in general wards. Our survey methodology relied on questionnaires, potentially leading to the preferential inclusion of younger, more educated patients and introducing a risk of selection bias. Thirdly, the outbreak in our study was attributed to the Omicron variant strain. SARS-CoV-2 infection and COVID-19 may have varying effects worldwide due to different health care systems. To date, there have been multiple variants of COVID-19, and these variants may exhibit varying levels of virulence and long-term consequences, potentially limiting the generalizability of our findings to later variant infections. Additionally, the non-specific nature of some post-COVID-19 sequelae complicates the differentiation between symptoms arising from the pandemic itself and those attributable to comorbidities, aging, or the broader societal effects of the pandemic experience.

## 6 Conclusion

This study suggests that MG patients are more likely to develop infections and have more severe symptoms compared to the general population. The severity of COVID-19 in MG patients correlates with the daily CS dosage and the frequency of myasthenia crises pre-COVID-19 (≥3). And MG patients are more likely to experience post-COVID-19 sequelae such as insomnia, myalgia, dizziness, cough, expectoration, and sore throat.

These findings provide valuable insights into the long-term health outcomes for MG patients post-COVID-19 infection and assist physicians in informing patients about the potential disease trajectory.

## Data Availability

The raw data supporting the conclusions of this article will be made available by the authors, without undue reservation.
